# Chronification of metastatic leiomyosarcoma in 9 lines of therapy by precision oncology: a case report and review of the literature

**DOI:** 10.3389/fonc.2025.1626478

**Published:** 2025-09-02

**Authors:** Christian R. Klein, Sebastian Koob, Verena Tischler, Annkristin Heine, Peter Brossart, Georg Feldmann, Karin Mayer

**Affiliations:** ^1^ Clinic of Internal Medicine III, Oncology, Hematology, Immune-Oncology and Rheumatology, University Hospital Bonn, Bonn, Germany; ^2^ Department of Orthopedics and Trauma Surgery, University of Bonn, Bonn, Germany; ^3^ Institute of Pathology, University Hospital Bonn, Bonn, Germany

**Keywords:** leiomyosarcoma, precision oncology, next-generation sequencing, molecular tumor boards, cancer chronification

## Abstract

Leiomyosarcoma is a malignant soft tissue tumor that still has a very poor prognosis in the metastatic stage, often lasting only several months. In addition to surgery and radiotherapy, the conventional treatment of this tumor entity is determined by chemotherapeutic regimes. Apart from anti-angiogenetically effective substances, hardly any targeted therapy options have been established. Here, we report the case of a 70-year-old man with metastatic leiomyosarcoma, who was able to be chronified by nine lines of oncological therapy over a period of four years, in addition to partial tumor resection and radiotherapy. The survival reported here is far greater than would be expected under approved standard therapy. Key to the long-term treatment of this patient was comprehensive pancancer panel sequencing (CCP, next-generation sequencing of genomic DNA) of the cancer tissue to search for molecular targets. This detected a loss-of-function mutation in a homologous recombination repair (HRR) gene, enabling treatment with the PARP inhibitor olaparib. Another special feature was the addition of the alkylating cytostatic agent temozolomide; the effectiveness of this combination therapy has so far only been shown for uterine leiomyosarcoma, but also proved to be an effective therapeutic strategy in the case of a male patient reported here. Despite high cumulative doses of previously applied chemotherapy, the targeted oncological treatment was tolerable and effective. The case report shows the high value of systematic molecular sequencing of cancer tissue and presentation in molecular tumor board for identification of molecular target structures for optimized palliative systemic therapy of metastatic leiomyosarcoma. In addition, the case report demonstrates that the combination therapy olaparib/temozolomide may also be an effective treatment approach for nonuterine leiomyosarcoma with HRR loss of function.

## Introduction

Leiomyosarcomas (LMS) are malignant soft tissue sarcomas (STS) in adults and exhibit high molecular heterogeneity. In addition to histopathological diagnosis, three molecular LMS subtypes can be distinguished by proteome clustering (1–3). Furthermore, soft tissue sarcoma subtypes can be characterized by DNA methylation signatures (4). Uterine leiomyosarcoma (uLMS) is distinguished as a separate entity from LMS of other locations due to divergent cytogenetics and gene expression patterns (5, 6). Metastatic LMS are currently treated primarily with chemotherapy; in addition to multi-targeted tyrosine kinase inhibitor (Multi-TKI) Pazopanib (7), there are hardly any targeted therapy options so far. Overall survival of metastasized leiomyosarcomas is poor, with a median survival time of less than two years (8–10). The LMS tumor microenvironment (TME) is special in terms of immuno-oncology; effective immune checkpoint inhibition (ICB) requires B-cell-rich tertiary lymphoid structures in soft tissue sarcomas (11); although the cellular composition in the LMS TME predicts ICB effectiveness (12), ICB has not yet been approved for treatment. Recently, HER2-directed CAR-T cell therapy has been reported for the treatment of refractory sarcomas (13, 14), whereas other cellular immunotherapies have not yet been established for this entity.

Here, we report on the treatment of a patient with metastatic LMS, which was chronified in 9 lines of therapy over a period of four years. Key to this was pancancer panel sequencing, which detected an HRR loss-of-function mutation that allowed PARP inhibition with olaparib. The combination of olaparib with temozolomide has so far only been established for uLMS, but it also proved to be effective and tolerable in the present case of retroperitoneal sarcoma in a male patient.

## Case presentation

**Figure 1:**
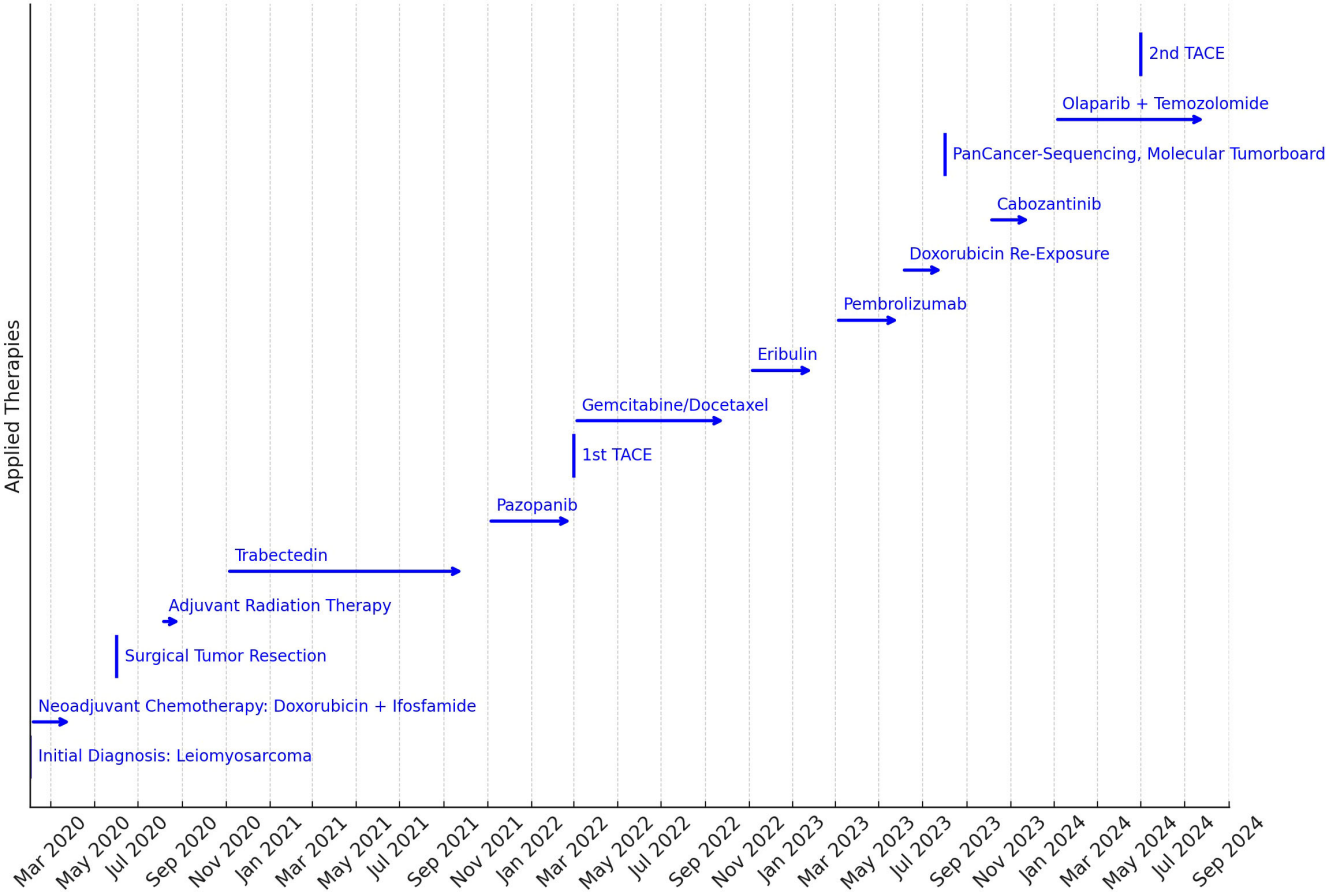
Timeline of the therapies applied. The length of the arrows indicates the duration of the treatments applied. The key to treatment was PanCancer sequencing and discussion in the Molecular Tumor Board to identify molecular target structures for targeted therapy in the third year of treatment.

In February 2020, a 70-year-old male patient with diffuse back pain was found to have a retroperitoneal mass (4x6x8 cm; height: L2 vertebral body) on a computed tomography (CT) scan. At that time, he was in a good general condition with an Eastern Cooperative Oncology Group (ECOG) performance status of 1. Subsequent PET-CT did not reveal any further malignant-typical, hypermetabolic structures. The patient was not aware of any other pre-existing conditions besides medically controlled arterial hypertension and a nodular goiter with hyperthyroid metabolism. CT-guided puncture of the mass yielded the diagnosis of a 75mm large, moderately differentiated, actively proliferating (Ki67 60-70%) LMS. Initially, the patient received three cycles of neoadjuvant chemotherapy with doxorubicin (75mg/m^2^) and ifosfamide (5g/m^2^) via a port system from 02-04/2020. During this treatment, neurotoxicity CTCAE III° occurred, which led to discontinuation of therapy. In 06/2020, a median laparotomy with retroperitoneal tumor extirpation with lymph node extirpation was performed. Pathologically, leiomyosarcoma was classified as pT2 pN1 (1/6, ece-), cM0, R2, L0, V0, Pn0, 2 + 2 + 1 according to FNCLCC, microsatellite-stable, stage II UICC/AJCC 2017. In 08-09/2020, adjuvant radiotherapy was applied (25x 2 Gy, total dose 50 Gy; boost: 16 Gy to the R2 region). In 10/20, CT showed pulmonary and osseous (LWK1) tumor progression. Trabectedin was subsequently administered as second-line therapy over 14 cycles from 11/20-10/21; due to WHO grade IV neutropenia and upper gastrointestinal bleeding with duodenitis, a dosage reduction to 60% was necessary during the course of treatment. In 11/21, there was osseous, muscular and bi-pulmonary tumor progression, as well as a new solitary liver metastasis, leading to a treatment switch to pazopanib (800mg/d). Due to persistent leuko- and thrombopenia, therapy had to be steadily reduced to 400mg/d in 02/2022. With further cancer progression in 03/2022, therapy was changed to gemcitabine/docetaxel (03-10/2022), as well as TACE of solitary liver metastasis. With pulmonary and osseous (T9) cancer progression in 11/2022, therapy was changed to eribulin. In 02/2023, renewed tumor progression under this treatment was detected, so that ICB with pembrolizumab was performed from 03-06/2023. This also led to tumor progression (lung, BWK9, liver, pancreas), so that in 06-08/2023 re-exposure to doxorubicin (mono, 2 cycles, dose-reduced 60mg/m^2^) was administered. In 08/2023, a PD with spinal canal infiltration at T9 was observed, making radiotherapy necessary in 09/2023 (T9: 30 Gy; boost of the intraspinal soft tissue component: 39 Gy). From 10-12/2023, the patient received cabozantinib (off-label use) after cost coverage was approved by health insurance. At the same time, comprehensive pancancer sequencing of biopsied tumor tissue was performed in 08/2023 (TruSight™ Oncology 500 assay: next-generation sequencing (NGS) of genomic DNA to search for mutations in 523 target genes and RNA for fusion analysis of 56 target genes) and followed by a discussion of the findings in the molecular tumor board. Molecular pathology findings from this diagnostic procedure are summarized in **Table 1**: In addition to an oncogenic loss-of-function mutation in TP53, the mutation c.1111 C>T in RAD51B, which codes for a translational stop codon and thus presumably induces premature translation termination with associated loss of function in HRR (homologous recombination repair) genes, was found. This finding was the basis for a further line of therapy in the event of recurrent cancer progression in 01/2024. Due to the loss-of-function mutation in HRR genes, therapy with olaparib in combination with temezolomide was started. Here (based on (15)), treatment was started with an initial dose of temozolomide 50mg/m^2^ + olaparib 2x200mg BID and steadily increased to a target dose of up to 75mg/m^2^ temozolomide (d1-7); olaparib 200mg bid (d1-7), cycle once every 21 days. With stable extrahepatic findings, second TACE of the hepatic metastases was performed in 05/2024 and tolerated without complications. Visualization of the timeline is shown in **Figure 1**. Until 09/2024, the patient was in an improved overall condition with stabilized disease findings without particular side effects due to this therapy. Noteworthy is the patient’s continued performance status (ECOG 2). Despite nine lines of therapy, he was still able to attend outpatient appointments independently with the aid of a wheelchair, which underscores his well-preserved quality of life.

**Table 1:** The table shows the therapy lines applied, start and end time of therapy, specific side effects observed, duration of application/progression-free survival in months and therapeutic mechanism of the substances.

Therapy Line and Substance	Start Date	End Date	Observed Side Effects	Duration of application/PFS (Months)	Best Response	Therapeutic Mechanism
1. Doxorubicin (75mg/m²), Ifosfamid (5g/m²)	02/2020	04/2020	Neurotoxicity (Grade III)	2	Neoadjuvant	DNA intercalation and inhibition of topoisomerase II (Doxorubicin), alkylating agent (Ifosfamide)
2. Trabectedin	11/2020	10/2021	Neutropenia (WHO °IV), gastrointestinal bleeding (Duodenitis)	11	Stable Disease (SD)	Inhibition of RNA polymerase II transcription
3. Pazopanib	11/2021	03/2022	Leuko- and thrombopenia	4	Stable Disease (SD)	Tyrosine kinase inhibitor
4. Gemcitabin/Docetaxel	03/2022	10/2022	None reported	8	Stable Disease (SD)	Antimetabolite (Gemcitabine), microtubule-targeting agent (Docetaxel)
5. Eribulin	11/2022	02/2023	None reported	3	Progressive Disease (PD)	Microtubule inhibitor
6. Pembrolizumab	03/2023	06/2023	None reported	4	Progressive Disease (PD)	Immune checkpoint inhibitor (PD-1)
7. Doxorubicin (mono)	06/2023	08/2023	None reported	2	Progressive Disease (PD)	DNA intercalation and inhibition of topoisomerase II
8. Cabozantinib	10/2023	12/2023	None reported	3	Stable Disease (SD)	Tyrosine kinase inhibitor (VEGFR, MET, RET)
9.Olaparib (200mg) Temozolomide (50 - 75mg/m^2^)	01/2024		None reported	9	Stable Disease (SD)	PARP inhibitor (Olaparib), alkylating agent (Temozolomide)

PFS, Progression-Free Survival; SD, Stable Disease; PD, Progressive Disease.

## Discussion

The case report of a metastasized LMS presented here is an remarkable example of cancer chronification in nine lines of therapy. The key to the treatment of this patient in the eighth and ninth lines of therapy was the identification of molecular targets in the cancer tissue using pancancer sequencing and discussion of the findings in the molecular tumor board.

### Discussion of lines of therapy

#### Initial chemotherapy

The patient’s initial treatment was carried out with a neoadjuvant chemotherapy of doxorubicin and ifosfamide, oncological resection and adjuvant radiotherapy. The combination therapy with doxorubicin and ifosfamide was chosen with the rationale of a neoadjuvant tumor reduction for oncological resection; however, in the case of a non-neoadjuvant indication, the combination of doxorubicin and ifosfamide showed no advantage over doxorubicin alone in terms of overall survival (OS) (8). Trabectedin and the multi-tyrosine kinase inhibitor (VEGFR, PDGFR, KIT) Pazopanib are established second- and third-line substances in the event of a relapse. When the disease progressed again, a therapy containing gemcitabine (gemcitabine + docetaxel) was chosen (16). The SARC-002 study showed higher remission rates and longer PFS in uLMS for this combination (17), but the TAXOGEM study, which recruited for uLMS and non-uLMS, showed no superiority over gemcitabine monotherapy (18). Subsequent therapy with eribulin can be considered as equally effective as dacarbazine (19), but it also did not show any longer-term disease control.

#### Immune checkpoint blockade

In the presented case, ICB with the PD1 inhibitor pembrolizumab was not effective. This is congruent with the results of the SARC028 study, in which none of the patients with LMS included in the study showed a response to therapy. Interestingly, a certain heterogeneity in the effectiveness of ICB in STS can be observed in principle, with response rates of up to 40% reported for undifferentiated pleomorphic sarcoma (5, 20). B-cell-rich tertiary lymphoid structures appear to play a crucial role in the effectiveness of ICB in STS (11). Furthermore, STS with MYC/MTORC1-activated epithelioid malignant cells and CLEC5A/SPP1+-M2-like immunosuppressive macrophages in the TME show greater responses to ICB (12). In the case presented here, previous chemotherapy exposure may have contributed to certain B-cell depletion and, consequently, to reduced ICB efficacy.

#### Multi-tyrosine kinase inhibitors: pazopanib, cabozantinib

The multi-tyrosine kinase inhibitor (multi-TKI) Pazopanib was approved as a second-line treatment for STS in the PALETTE study (7). In the case described here, pazopanib achieved disease control for 4 months with 4.6 months of progression-free survival (PFS) in the PALETTE study. The multi-TKI cabozantinib (inhibits MET, VEGF, AXL, RET, ROS1, TYRO3, MER, KIT, TRKB, FLT3, TIE-2) was used in 8th line of therapy. The drug has not yet been approved for STS, and previous studies report 6-month PFS of 49% for STS (21) and 33% for osteosarcoma (22). In the case presented here, disease control was achieved for 3 months despite prior chemotherapy, immune checkpoint and multi-TKI therapy. The case shows that despite previous multi-TKI therapy, consecutive treatment with another multi-TKI in LMS can have a certain therapeutic effect. Further case reports on TKI therapy in STS can be found in (23–27). Reviews on TKI in sarcomas are (28–35).

### Pancancer sequencing, molecular tumor board, olaparib/temozolomide

Key to the further treatment of this case report was pancancer sequencing and presentation of the patient to a molecular tumor board. Comprehensive genomic profiling (CGP) was carried out using the TruSight™ Oncology 500 Assay, in which 523 cancer-associated genes were tested for single nucleotide variants (SNV), insertions and deletions (indels), copy number variations (CNV), tumor mutational burden (TMB, [mut/Mb]), and microsatellite instability (MSI). The results of the mutation diagnostics are summarized in **Table 2**. Furthermore, a TMB-low status (5.98 mutations/Mb) was found, as well as microsatellite instability (MSS, 7/9 markers stable, 1 marker not evaluable). The mutation c.743G>A in TP53 was assessed as pathogenic in the Clinvar mutation database. In the TP53 mutation database IARC, the variant is listed as non-functional based on transcriptional transactivation assays in yeast (36), and there are currently no targeted treatment options in the context of approval or off-label use. The c.1111C>T mutation in RAD51B creates a translational STOP codon and thus presumably leads to premature termination of translation and a concomitant loss of function of the encoded protein. This loss-of-function mutation in HRR (homologous recombination repair) genes was one rationale for off-label therapy with the PARP inhibitor olaparib. The mutations in PDGFRA and PMS2 were evaluated in ClinVar as variants of uncertain significance, hence, based on the current data, no conclusive statement on the clinical relevance of the two sequence variants was possible. However, they were classified as presumably benign rare variants. On the basis of the pancancer sequencing, the molecular tumor board confirmed possible treatment with a PARP inhibitor such as olaparib or rucaparib. These agents are approved for treatment of BRCA-mutated [germline and/or somatic] platinum-sensitive relapsed high-grade serous epithelial ovarian cancer. The changes presumably lead to nonsense-mediated mRNA decay or to premature termination of translation and a concomitant loss of function of the respective encoded proteins. PARP inhibition in loss-of-function mutations in HRR genes has already been described as a principal therapeutic option for sarcomas (37). In the Nira-Panc study, the effectiveness of a monotherapy with the PARP inhibitor niraparib had already been investigated in first-line progressive pancreatic cancer with a RAD51B mutation, among others (6-month PFS rate of 40%, median PFS of 4.4 months, median OS of 9.1 months) (38). There is initial experimental evidence for the effectiveness of niraparib in HRR-deficient STS (39).

**Table 2:** Findings of comprehensive pancancer sequencing as part of the molecular tumor board in 08/2023.

Gene	Mutation	Interpretation	Therapeutic relevance
TP53	c.743G>A p.Arg248Gln	oncogenic, loss of function	No targeted therapies for TP53 alone
RAD51B	c.1111C>T p.Gln371*	truncating, likely loss of function	Relevant - HRR gene (PARP inhibitor sensitivity)
PMS2	c.869G>A p.Gly290Arg	uncertain, likely benign	Not therapeutically relevant
ATRX	c.4690G>T p.Gln1564Glu	variant of uncertain significance	Unclear significance
IRS2	c.2174G>T p.Gly725Val	variant of uncertain significance	Unclear significance
PDGFRA	c.1285G>A p.Gly429Arg	uncertain, likely benign	Not therapeutically relevant
CARD11	c.3004A>T p.Thr1002Ser	variant of uncertain significance	Unclear significance
FGFR3	c.112G>A p.Val38Ile	variant of uncertain significance	Unclear significance

Due to the fundamental possibility of a targeted therapy option with a PARP inhibitor, olaparib with the alkylating cytostatic agent temozolomide was chosen in the ninth line of therapy. During the molecular tumor board discussion, the identified RAD51B loss-of-function mutation was recognized as the primary actionable target, conferring a “BRCAness” phenotype and predicting sensitivity to PARP inhibitors. Other alterations were also considered: the pathogenic TP53 mutation lacks a targeted therapy, and the PDGFRA and PMS2 variants were classified as VUS (variants of uncertain significance), precluding them as a basis for therapy.

**Figure 2:**
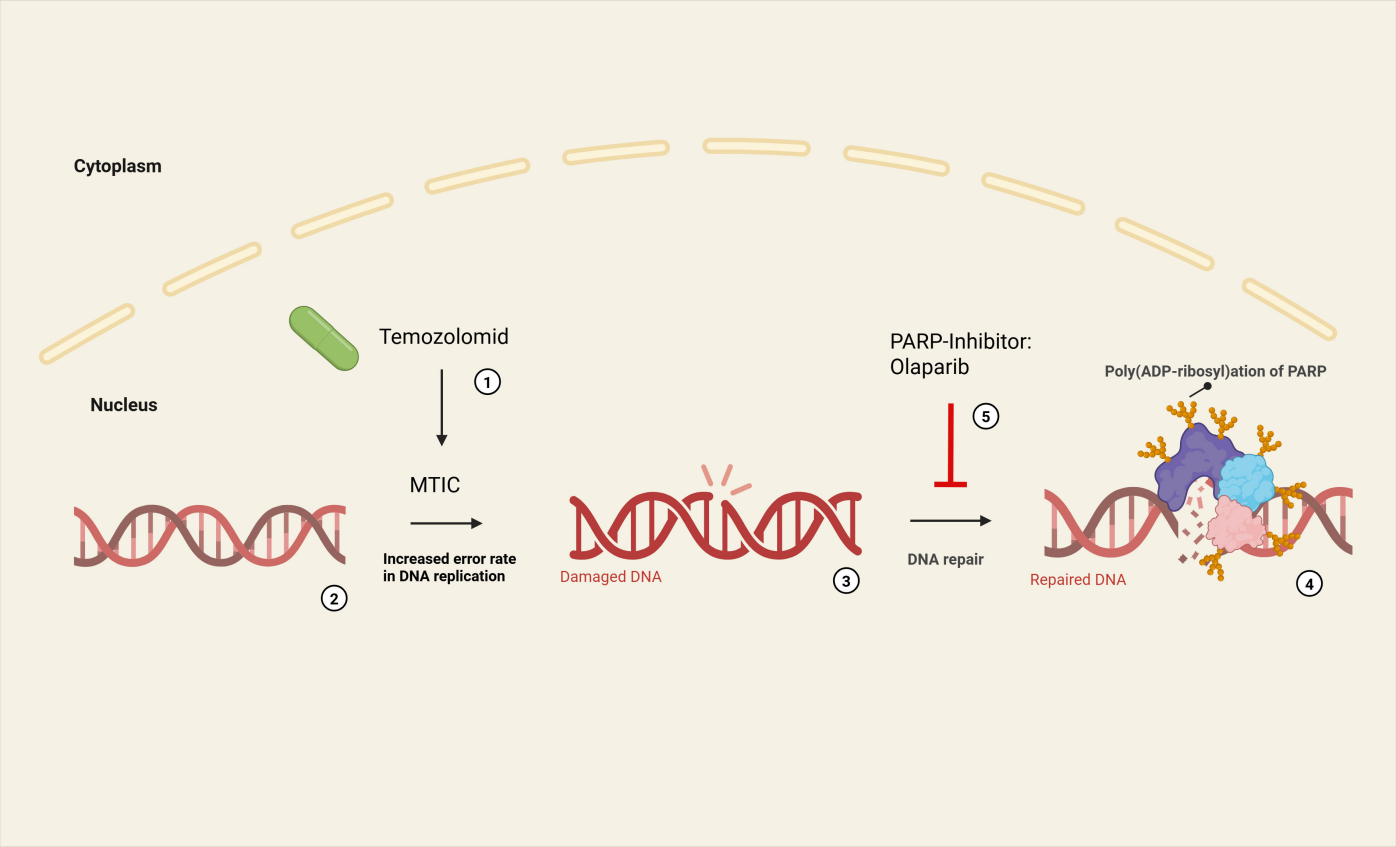
Synergistic effect of temozolomide and olaparib (9th line of therapy after pancancer sequencing and molecular tumor board): Temozolomide breaks down at neutral pH to form its active metabolite methyltriazenoimidazole carboxamide (MTIC) (1). MTIC methylates DNA bases (6-O-methylguanine, N7-methylguanine, N3-methyladenine) (2). This leads to an increased error rate in DNA replication (3) as part of the cellular repair mechanisms (MMR). Physiologically, poly (ADP-ribose) polymerases (PARP) recognize DNA-SSB and catalyze a poly (ADP-ribose) modification that enables a BER (by DNA ligase III and DNA polymerase β) (4). The PARP inhibitor olaparib blocks enzymatic activity of PARP, thereby preventing auto-PARylation and recruitment of other repair proteins (5). Furthermore, DNA-bound PARP can cause DNA damage and thus induce apoptosis itself (‘PARP trapping’).

Several therapeutic strategies for the RAD51B mutation were weighed. Platinum-based chemotherapy, a potential option for HRR-deficient tumors, was considered but deferred due to the patient’s extensive pretreatment and the desire for a less toxic, oral regimen. PARP inhibitor monotherapy was another key option. The rationale for the combination therapy olaparib/temozolomide was based on preclinical data (40) and the results of a phase II study for RAD51-recombination-deficient uLMS (15). However, PARP inhibition for other sarcoma entities has so far only been described in a very small number of case reports (41, 42). The synergistic effect of temozoloid and olaparib is visualized in **Figure 2**.

### uLMS versus non-uLMS

The case report presented here exemplifies efficacy of the combination therapy olaparib/temozolomide in non-uLMS of a male patient, which has so far only been investigated for uLMS. The response to therapy more than doubled the efficacy of the four previous lines of therapy over an observation period of 9 months. The case report shows that this therapeutic strategy for uLMS can also be effective for non-uLMS and that the separate cytogenetic profile of uLMS (5, 6) does not necessarily have to influence molecularly targeted therapy. In this sense, the pathological LMS differentiation (uLMS versus non-uLMS) does not reflect the clinical-therapeutic similarities. The existence of molecular target structures, as detected in the pancancer sequencing, was the sole decisive factor for therapy selection here and shows that even the higher resolution of a molecular pathological classification cannot necessarily predict optimal therapeutic strategies.

The HRR deficiency in RAD51B found in this case confers BRCAness, i.e. susceptibility to DNA repair defects in the homologous recombination repair pathway (HRR), similar to tumors with mutations in BRCA1/2 genes. Cancer cells with BRCAness frequently show sensitivity to PARP inhibitors, since inhibition of poly(ADP-ribose) polymerases (PARPs) inhibits DNA single-strand break (SSB repair) so that, in the presence of HRR deficiency, double-strand breaks (DSB) accumulate, leading to cell death (‘synthetic lethality’ (43)). It is noteworthy that HR deficiency and the Alexandrov COSMIC mutation signature AC3 (associated with HRD) are found in the majority of cases in whole exome (WES) and transcriptome sequencing of LMS and predict olaparib sensitivity in a dose-dependent manner (44, 45). uLMS are more likely to show HRD than non-uLMS and the proportion of HR-deficient uLMS is among the highest of all tumor types in TCGA (46); uLMS show the highest rate of homozygous BRCA2 deletion in comparative analyses (47); our case report also demonstrates BRCAness in non-uLMS and motivates the possibility of including the option of PARP inhibition in non-uLMS in therapeutic considerations.

### Limitations

The reported long therapeutic response under olaparib/temozolomide was probably not reduced by previous therapies in the case discussed. However, due to B-cell-dependent ICB effectiveness in STS, it can be assumed that previous B-cell-depleting chemotherapy has reduced ICB efficacy. Prospective studies are needed in the future for general recommendations, but the possible negative influence of previous B-cell-depleting therapies on the effectiveness of ICB should be kept in mind when treating sarcomas.

Chemotherapy re-exposure with doxorubicin (mono) was ineffective compared to primary application (in combination with ifosfamide), which is why treatment was switched to cabozantinib after only two months. The case reported here suggests that tumor re-challenging with doxorubicin as a first-line substance is not effective. Chemotherapy re-challenge in STS has been very poorly studied in the scientific literature to date (48).

In principle, re-biopsies and/or liquid biopsy during the course of therapy and a new pancancer sequencing to detect escape mutations and possibly new therapeutic target structures in the course of cancer evolution would be useful (49, 50). At the time of completion of the case report, the patient showed stable disease under the 9th line of therapy, which is why no re-sequencing had yet been performed.

Due to high variant allele frequency (VAF) for TP53 and sarcoma as a leading malignancy for Li-Fraumeni syndrome (LFS), germline testing could be considered. However, this was not carried out due to the patient’s refusal.

### Conclusion and outlook

The case report documents chronification of metastatic non-uLMS in 9 lines of therapy and, to the best of our knowledge, has not yet been reported in the literature. Survival as reported here is far greater than might be expected under standard therapy. Despite high cumulative doses of previous chemotherapy, targeted therapies were effective and well tolerated. The key to the treatment was pancancer sequencing, which revealed a RAD51B mutation and thus the possibility of PARP inhibition. The chosen combination therapy olaparib/temozolomide has so far only been investigated for uLMS, but it also showed good efficacy in the case presented here despite multiple, less effective previous lines of therapy. Therapeutic strategies for uLMS can be effective in non-uLMS despite their own molecular pathology, and the disjunctive cytogenetic profile of uLMS is not necessarily a prerequisite for this therapy. This also motivates the pursuit of pancancer sequencing and discussion in a molecular tumor board in the case of late-stage disease. In the case of metastasized LMS, pancancer sequencing and early discussion in a molecular tumor board can be recommended.

## Data Availability

All data supporting the findings of this study are available within the article.

## Glossary

AC3: Alexandrov COSMIC Mutational Signature 3
BER: Base Excision Repair
BRCA: Breast Cancer Gene
CAR-T: Chimeric Antigen Receptor T-Cell
CCP: Comprehensive Cancer-Panel Sequencing
CGP: Comprehensive Genomic Profiling
CLEC5A: C-Type Lectin Domain Family 5 Member A
CNV: Copy Number Variation
CT: Computed Tomography
DNA-DSB: DNA Double-Strand Break
DNA-SSB: DNA Single-Strand Break
ECOG: Eastern Cooperative Oncology Group
FLT3: Fms-like Tyrosine Kinase 3
FNCLCC: Fédération Nationale des Centres de Lutte Contre le Cancer
GAS6 Receptor (AXL): Growth Arrest-Specific 6 Receptor
Gy: Gray (unit of radiation dose)
HER2: Human Epidermal Growth Factor Receptor 2
HRD: Homologous Recombination Deficiency
HRR: Homologous Recombination Repair
IARC: International Agency for Research on Cancer
ICB: Immune Checkpoint Blockade
Indels: Insertions and Deletions
KIT: KIT Proto-Oncogene (CD117, receptor tyrosine kinase)
LFS: Li-Fraumeni Syndrome
LMS: Leiomyosarcoma
LWK: Lumbar Vertebra
MER: MERTK Proto-Oncogene, Tyrosine Kinase
MET: Hepatocyte Growth Factor Receptor Protein
MMR: Mismatch Repai: MSI, Microsatellite Instability
MSS: Microsatellite Stability
MTIC: Methyltriazenoimidazolcarboxamid
MTORC1: Mechanistic Target of Rapamycin Complex 1
Multi-TKI: Multi Tyrosine Kinase Inhibitor
MYC: Myc Proto-Oncogene Protein
NGS: Next-Generation Sequencing
OS: Overall Survival
PARP: Poly (ADP-Ribose) Polymerase
PFS: Progression-Free Survival
PD: Progressive Disease
PD1: Programmed Cell Death Protein 1 (an immune checkpoint protein)
PDGFR: Platelet-Derived Growth Factor Receptor
PDGFRA: Platelet-Derived Growth Factor Receptor Alpha
PET-CT: Positron Emission Tomography - Computed Tomography
PMS2: Postmeiotic Segregation Increased 2 (DNA mismatch repair gene)
RAD51B: RAD51 Paralog B (involved in HRR)
RET: REarranged during Transfection (proto-oncogene receptor tyrosine kinase)
ROS1: ROS Proto-Oncogene 1, Receptor Tyrosine Kinase
SNV: Single Nucleotide Variant
SPP1: Secreted Phosphoprotein 1
STS: Soft Tissue Sarcoma
TACE: Transarterial Chemoembolization
TCGA: The Cancer Genome Atlas
TIE-2: TEK Receptor Tyrosine Kinase (also known as Tunica Internal Endothelial Cell Kinase)
TMB: Tumor Mutational Burden
TME: Tumor Microenvironment
TRKB: Tropomyosin Receptor Kinase B
TYRO3: Tyrosine-Protein Kinase Receptor 3
UICC/AJCC: Union for International Cancer Control/American Joint Committee on Cancer
uLMS: Uterine Leiomyosarcoma
VUS: Variant of Uncertain Significance
VAF: Variant Allele Frequency
VEGF: Vascular Endothelial Growth Factor
VEGFR: Vascular Endothelial Growth Factor Receptor
WES: Whole Exome Sequencing
